# Comparison of different trapping methods to collect malaria vectors indoors and outdoors in western Kenya

**DOI:** 10.1186/s12936-024-04907-0

**Published:** 2024-03-16

**Authors:** Jackline Kosgei, John E. Gimnig, Vincent Moshi, Seline Omondi, Daniel P. McDermott, Martin J. Donnelly, Collins Ouma, Bernard Abong’o, Eric Ochomo

**Affiliations:** 1 Entomology Section, Centre for Global Health Research, Kenya Medical Research Institute, P.O. Box 1578-40100, Kisumu, Kenya; 2 Department of Vector Biology, Liverpool School of Tropical Medicine, Pembroke Place, Liverpool, L3 5QA UK; 3 Department of Biomedical Sciences and Technology, Maseno University, Maseno, Kenya; 4 Division of Parasitic Diseases and Malaria, Centers for Disease Control and Prevention, Atlanta, GA 30333 USA

**Keywords:** UV light trap, Human landing catches, *Anopheles*, Trapping methods

## Abstract

**Background:**

Vector surveillance is among the World Health Organization global vector control response (2017–2030) pillars. Human landing catches are a gold standard but difficult to implement and potentially expose collectors to malaria infection. Other methods like light traps, pyrethrum spray catches and aspiration are less expensive and less risky to collectors.

**Methods:**

Three mosquito sampling methods (UV light traps, CDC light traps and Prokopack aspiration) were evaluated against human landing catches (HLC) in two villages of Rarieda sub-county, Siaya County, Kenya. UV-LTs, CDC-LTs and HLCs were conducted hourly between 17:00 and 07:00. Aspiration was done indoors and outdoors between 07:00 and 11:00 a.m. Analyses of mosquito densities, species abundance and sporozoite infectivity were performed across all sampling methods. Species identification PCR and ELISAs were done for *Anopheles gambiae* and *Anopheles funestus* complexes and data analysis was done in R.

**Results:**

*Anopheles* mosquitoes sampled from 608 trapping efforts were 5,370 constituting 70.3% *Anopheles* *funestus *sensu lato (*s.l.*), 19.7% *Anopheles coustani* and 7.2% *An. gambiae s.l*. 93.8% of *An. funestus s.l.* were *An. funestus *sensu stricto (*s.s*.) and 97.8% of *An. gambiae s.l.* were *Anopheles* *arabiensis*. Only *An. funestus* were sporozoite positive with 3.1% infection prevalence. Indoors, aspiration captured higher *An. funestus* (mean = 6.74; RR = 8.83, *P* < 0.001) then UV-LT (mean = 3.70; RR = 3.97, *P* < 0.001) and CDC-LT (mean = 1.74; RR = 1.89, *P* = 0.03) compared to HLC. UV-LT and CDC-LT indoors captured averagely 0.18 *An. arabiensis* RR = 5.75, *P* = 0.028 and RR = 5.87, *P* = 0.028 respectively. Outdoors, UV-LT collected significantly higher *Anopheles* mosquitoes compared to HLC (*An. funestus*: RR = 5.18, *P* < 0.001; *An. arabiensis*: RR = 15.64, *P* = 0.009; *An. coustani*: RR = 11.65, *P* < 0.001). *Anopheles funestus* hourly biting indoors in UV-LT and CDC-LT indicated different peaks compared to HLC.

**Conclusions:**

*Anopheles funestus* remains the predominant mosquito species. More mosquitoes were collected using aspiration, CDC-LTs and UV-LTs indoors and UV-LTs and CD-LTs outdoors compared to HLCs. UV-LTs collected more mosquitoes than CDC-LTs. The varied trends observed at different times of the night suggest that these methods collect mosquitoes with diverse activities and care must be taken when interpreting the results.

**Supplementary Information:**

The online version contains supplementary material available at 10.1186/s12936-024-04907-0.

## Background

The Global Vector Control Response 2017–2030 (GVCR) provides a framework to enhance vector control through improved capacity and surveillance, and through better coordination and integrated action across sectors and diseases. One of the four pillars of this strategy is enhanced vector surveillance [1]. Robust vector surveillance is critical for monitoring currently recommended vector control tools as well as to evaluate novel control strategies [2]. The objectives for vector surveillance of the World Health Organization (WHO) include: characterizing receptivity (to evaluate vector presence and density to enable selection and stratification of interventions), tracking of malaria vector densities (for selection and timing of vector control deployment by biting time or seasonality of transmission), monitoring of insecticide resistance (IR) for selecting insecticides to be used by programmes, and identifying other threats to vector control efficacy including detecting gaps in intervention coverage [3, 4]. However, the range of entomological surveillance methods currently available may lack the sensitivity to detect subtle changes in vector behaviours or may not be adequate to evaluate the performance of novel vector control tools that may target a greater diversity of adult mosquito behaviours [5, 6].

The most common vector control tools—long-lasting insecticidal nets (LLINs) and indoor residual spraying (IRS)—target indoor biting mosquitoes that are most active when people are in bed (LLINs) or that spend time resting on walls inside the house (IRS). The effectiveness of these vector control tools is threatened by changes in vector biting and resting behaviour and the diversity of their vectorial system [7]. For example, some mosquitoes either bite outdoors [8] or indoors at times that people are not under the protection of their bed nets [9]. LLINs and IRS have a direct impact on vector bionomics [10] and, historically, have been monitored using human landing catches (HLC), CDC light traps, pyrethrum spray catches, and aspiration techniques.

The HLC technique is a method in which human volunteers sit indoors and/or outdoors and collect mosquitoes that land on them throughout the night. It is considered the gold standard for sampling host-seeking mosquitoes [11] and for the estimation of entomological exposure rates [12, 13] for the evaluation of vector control interventions, and for the study of mosquito behaviour [12, 14, 15]. However, HLC is labour-intensive, exposes human collectors to potentially infectious mosquito bites, and is subjected to collector bias [16]. Other surveillance tools like light traps, pyrethrum spray catches and aspiration are less expensive and can be implemented more widely, but the information they provide is generally limited to indoor abundance. These methods either cannot be implemented outdoors at all or are thought to be inefficient in capturing mosquitoes outdoors. Furthermore, they generally do not provide information on mosquito behaviour, particularly the time and location of mosquito biting. Additionally, in the context of evaluating novel vector control tools, it is prudent assess surveillance tools that can provide information on subtle changes in mosquito behaviour, hence the inclusion of two outdoor locations in this study.

This study evaluated CDC light traps (CDC-LT), UV light traps (UV-LT) and Prokopack® aspiration (hereafter referred to as aspiration) against HLC conducted either inside or outside houses as potential tools for monitoring mosquito populations.

## Methods

### Study area

The study was conducted in Memba (− 0.16118, 34.36639) and Mabinju (− 0.17966, 34.37003) villages in Rarieda sub-county, Siaya county, western Kenya. Residents of the area live in scattered compounds which consist of an average of 3 houses occupied by closely related family members and interspersed with farmlands. The area immediately around the house structures is usually delineated from the surrounding farmland by a fence or hedges. The area experiences intense, year-round malaria transmission [17] with *Plasmodium falciparum* as the predominant malaria parasite species and *Anopheles funestus, Anopheles arabiensis* and *Anopheles gambiae *sensu stricto (*s.s*.) the main vectors [18, 19]. Historically, malaria transmission in western Kenya was very high with an estimated 300 infectious bites per person per year in the late 1980s and early 1990s [20]. Transmission has declined substantially since then, largely due to the scale up of insecticide treated nets through mass distributions targeting universal coverage (1 net for every 2 people) supplemented with routine distribution to pregnant women and children < 1 year [21]. However, the burden of malaria remains high with parasite prevalence at 19% in children aged 6 months to 14 years in the region [22]. Additionally, the deployment of malaria vector control tools such as ITNs has been accompanied by shifts in vector populations in this region beginning with the near complete disappearance of *An. funestus* [23], followed by a decline in *An. gambiae s.s* [19]*.* and a return of *An. funestus* [18]. Additionally, deployment of anti-vector interventions may lead to adaptive modifications in vector behavioural patterns [24–27]. These shifts in mosquito populations and vector behaviours necessitate frequent evaluation of trapping tools.

### Mosquito trapping methods

#### Light traps

CDC light traps (CDC-LTs) (model 512) and UV light traps (UV-LTs) (model 912) (John W. Hock Company, Gainesville, Florida, U. S. A), without artificial attractants, were used. The CDC light trap uses an incandescent light, while the UV light traps are similar to CDC-LTs in design but with an ultraviolet light bulb. The traps were installed by hanging them approximately 1.5 m above the ground either indoors or outdoors. The indoor traps were placed next to a person sleeping under a bed net whereas the outdoor traps were placed either 10 m from the structure (referred to hereafter as outdoors close) or 10 m outside the compound (referred to hereafter as outdoors far) where indoor sampling was conducted. The outdoor traps were not baited and relied only on source from the CDC and UV light traps to attract mosquitoes. All light traps were powered by a rechargeable 12-V battery and were switched on at 17:00. Collection cups for the traps were replaced every hour of the night by field staff in all instances of outdoor traps and a subset of indoor light traps. Twelve light traps out of 163 indoor collections were not picked from houses during hourly trapping as the compound owners refused entry after they had gone to bed. In those houses, light traps were collected at 07:00 a.m. the next morning. Data analysis from these houses included hourly mosquito activity up to the last time entry was granted; the rest of the collections till morning were excluded in the analysis of hourly biting but aggregate numbers of mosquitoes collected throughout the night were included in comparisons of trap efficiency.

#### Human landing catches

To reduce the risk of transmission of Covid-19, collectors were recruited from the compounds in which they lived. Collectors were males above the age of 18 years, organized into teams comprised of 6 volunteers. The team of six volunteers per compound were split into one indoor and two outdoor locations and they worked in two shifts. The first shift ran from 17:00 until 00:00 p.m. when the next team took over until 07:00 a.m. The volunteers were trained in HLC and provided with a flashlight, a mouth aspirator, mosquito collection cups and a hurricane lamp. The hurricane lamp was placed on the ground, approximately 1 m from the HLC collectors and turned as low as possible to allow for observation of mosquitoes landing on the collector’s legs [28]. The collectors sat on a chair with their legs exposed from foot to knee and captured mosquitoes as soon as they landed on the exposed leg [29]. Collections were conducted over 45 min within each hour with a 15-min break to allow collectors to rest and to change collection cups. Each hour’s collection was kept separately in labelled paper cups with the labels bearing unique hourly codes generated by the tablet and taking into account the village code, house number, collection method, collection location, collection day and collection time. The date of collection was also written on the paper cups. Supervisors were assigned to coordinate the collection activities and ensure volunteers were consistently engaged in mosquito collections throughout each collection night. HLC data was collected using tablets, with the forms programmed in CommCare® (Dimagi, Inc, Massachusetts, USA).

#### Resting collections (Aspiration)

Prokopack aspirators (Model 1419, John W Hock Company, Gainesville, Florida, USA) were used to collect mosquitoes resting indoors and outdoors from 07:00 to 11:00 a.m. A total of ten sleeping structures from different compounds nearest to the light trap and HLC houses were conveniently selected for aspiration. Sampling was done by moving the aspirator along the walls and the roof, in dark corners, and underneath furniture in the house to collect indoor resting mosquitoes for 10 min in each structure. Outdoor sampling was performed by aspirating from clay pots and other water collection containers that were already present in the compound and located within 5 m of each sampled house [30]. After every collection, the samples were released into an adult mosquito cage for sorting. The sampled mosquitoes from each collection were transferred to labeled paper cups per collection separating the outdoor and indoor catches. All aspirations were conducted by trained entomology staff for exactly 10 min per structure, timed using a stopwatch.

### House selection and rotation scheme

A community household survey was conducted in the two villages outlined in the months of September to October 2020. Prior to the start of the study, compound selection was done and houses with similar housing characteristics such as roof type, wall type and open eaves were recruited to participate in the study. Compounds from which mosquitoes were to be collected each night were randomly selected from the database of houses that had been identified and in the event that there were more than one inhabited houses in the compound, the primary house of the head of household was selected. A total of 160 houses were eligible for sampling during the study period. Different compounds were selected every night where the HLC, CDC-LT and UV-LT were rotated among 10 compounds in each village following a rotation schedule such that each house was sampled by each collection method the same number of times by the end of the study period. Aspiration was conducted the morning preceding HLC, CDC-LT and UV-LT in 10 compounds but close to those sampled the previous night. Each compound had only one trap type placed in three different positions: indoors, outdoors close and outdoors far. These locations were selected to enable a comprehensive assessment of the most efficient trapping method when collecting natural sugar fed mosquitoes that were likely to be captured as has been described in detail in a separate article [31]. The mosquito collections were done for 4 days every week over the two months’ study duration.

### Mosquito processing

Samples were transported in cooler boxes to the field laboratory in Lwak, Asembo for morphological assessment. Entomology field supervisors and a driver collected the paper cups with mosquitoes from the HLC collectors and light trap collection cups every hour of the night and placed them in cooler boxes containing ice packs for transport to the field laboratory. Upon reception at the field laboratory, the samples were immobilized by freezing at − 20 °C for a period of 10 min and for longer storage prior to processing. In the case of power outages or when the number of samples received from the field overwhelmed the available freezer space, mosquitoes were immobilized by exposing to chloroform in a killing jar for 1 min. The mosquitoes were separated by species, sex, and the abdominal status (unfed, half-fed, fed and gravid) for females and numbers collected per trap recorded**.** The mosquitoes were identified morphologically using taxonomic keys [32] to differentiate between *An*. *funestus* s.l. and *An. gambiae* s.l. and other secondary malaria vectors.

### Molecular assays

Polymerase chain reaction (PCR) was used to differentiate between mosquitoes of the *An. gambiae* species complex following the protocols described by Scott et al*.* [33] and between sibling species of the *An. funestus* complex using the protocols described by Koekemoer et al*.* [34]. All non-amplified samples were processed twice and the samples that were morphologically identified as *An. gambiae,* but failed to amplify were run using *An. funestus* primers and vice versa. Sporozoite infection rates were determined by enzyme linked immunosorbent assay (ELISA) using the protocol adapted from Wirtz et al*.* as described in the MR4 Methods in *Anopheles* Research [35, 36].

### Data analysis

Vector abundance was assessed using descriptive statistics (means, SD, proportions, and 95% CI). Separate analyses of trap comparisons were conducted on the three most common female species collected: *An. funestus *sensu lato (s.l.), *An. gambiae s.l.* and *Anopheles coustani s.l*. Aggregated numbers of mosquitoes collected each night were estimated for the primary analyses. For HLCs, no adjustments were made for the fact that collectors were operating for only 45 min within each hour. Since the data were over-dispersed, generalized linear mixed models (GLMM) using Template Model Builder (glmmTMB) were fitted using negative binomial distribution for analysis of daily mosquito numbers by various collection methods. Daily numbers of female *Anopheles* mosquitoes were assessed as a function of collection method as a fixed effect while collection compound and collection day were treated as random factors apart from when evaluating *An. arabiensis* outdoors where mosquito counts variation was not sufficient with both collection compound and collection day as random effect. Only collection compound was treated as a random effect while modelling *An. arabiensis* outdoors. Pairwise comparisons of the mean numbers of each *Anopheles* species collected by the different trapping methods were done by Tukey’s test. For assessment of hourly trap catches, data only included structures that had at least 12–14 collections during the night; structures/nights that did not achieve this threshold were excluded from these analyses of hourly collections. All data analyses were performed using R statistical software version 4.1.2 while all figures and graphs were fitted using ggplot2 package in R. Statistical significance level was set at α = 0.05.

## Results

### Abundance of *Anopheles* mosquitoes

A total of 5,370 male and female *Anopheles* mosquitoes were sampled during the study period from a total of 608 trapping efforts: CDC-LT (165), UV-LT (152), aspiration (158) and HLC (133). *Anopheles funestus* constituted more than half (n = 3780; 70.4%) of the sampled *Anopheles* mosquitoes with the rest being *An. gambiae s.l.* (n = 385; 7.2%), *An. coustani* (n = 1061; 19.7%) and other *Anopheles* species (n = 144; 2.7%) including *Anopheles pharoensis* (n = 120), *Anopheles rufipes* (n = 16), *Anopheles gibbinsi* (n = 5), *Anopheles maculipalpis* (n = 1), *Anopheles chrysti* (n = 1), and *Anopheles tenebrosus* (n = 1). Only *An. rufipes* (n = 3), *An. pharoensis* (n = 1) and *An. parensis* (n = 1) of the secondary Anophelines, other than *An. coustani* were collected indoors; the rest were trapped outdoors (Table 1). A total of 3,562 female mosquitoes were collected during the study (2169 *An. funestus*, 284 *An. gambiae* s.l., 973 *An. coustani*, 136 other *Anopheles*). All subsequent analyses included only females.

**Table 1:** Summary of mosquito numbers collected by each trapping method

Species	Trapping location	CDC-LT		UV-LT		HLC		Resting collections		Total Females	Total Males	Total *Anopheles*
		Females	Males	Females	Males	Females	Males	Females	Males			
*An. funestus*	Indoor	249	308	456	560	61	28	997	567	1763	1463	3777
	Outdoor	125	42	248	82	26	2	7	19	406	145	
*An. gambiae*	Indoor	26	15	51	16	2	0	53	28	132	59	385
	Outdoor	30	9	99	25	1	0	15	15	152	50	
*An. coustani*	Indoor	38	2	9	4	5	0	0	0	52	6	1061
	Outdoor	304	34	577	37	20	0	20	11	921	82	
Other *Anopheles* Species*	Indoor	0	4	0	0	0	0	0	0	0	4	147
	Outdoor	27	3	107	4	2	0	0	0	136	7	

^*^Other *Anopheles* species included: *An. pharoensis* (N = 120), *An. rufipes* (N = 16), *An. gibbinsi* (N = 5), *An. parensis* (N = 3), *An. maculipalpis* (N = 1), *An. chrysti* (N = 1), and *An. tenebrosus* (N = 1). Only *An. rufipes* (N = 3) and *An. parensis* (N = 1) were trapped indoors. The rest were trapped outdoors

A proportion of the sampled mosquitoes (51% of *An. funestus* and 53% of *An. gambiae*) were processed for species identification by PCR and sporozoite detection using ELISA. Out of the 1760 *An. funestus s.l.* samples analysed by PCR, 1650 (93.8%) were confirmed to be *An. funestus* s.s*.* and 45 (2.6%) *An. leesoni,* while 65 (3.7%) did not amplify. A total of 214 *An. gambiae* s.l*.* were processed through PCR out of which 209 (97.8%) were confirmed to be *An*. *arabiensis* and the remaining 5 (2.3%) samples did not amplify. A sample of the three predominant species *An. funestus* s.l. (862/2169, 39.7%)*, An. arabiensis* (168/284, 59.2%) and *An. coustani* (358/973, 36.8%) were analysed for *P. falciparum* sporozoite infection. Only *An. funestus* samples were positive, with a species specific sporozoite infection prevalence of 3.1% (27/862).

### Comparison of mean numbers of *Anopheles* mosquitoes caught per trapping method each night/day

The average number of mosquitoes collected indoors each night by HLC was 0.97 for *An. funestus*, 0.03 for *An. arabiensis*, and 0.08 for *An. coustani*. When compared to indoor HLC, indoor aspiration method captured the highest number of *An. funestus* with a mean of 6.74, (RR = 8.83, 95% CI 4.72–16.52, p < 0.001) followed by indoor UV-LT with a mean of 3.70, (RR = 3.97, 95% CI 2.28–6.92, p < 0.001) then indoor CDC-LT with a mean of 1.74 (RR = 1.89, 95% CI 1.07–3.34, p = 0.03). Compared to HLC, significantly higher numbers of *An. arabiensis* were collected indoors by UV-LT (RR = 5.87, 95% CI 1.22–28.34) and CD-LT (RR = 5.75, 95% CI 1.20–27.48) with a mean of 0.18 each, followed by aspiration and HLC, although these were not statistically different in pairwise comparisons. For *An. coustani*, the CDC-LT collected the highest number of mosquitoes indoors mean of 0.29 although this difference was not statistically different compared to HLC (RR = 2.01, 95% CI 0.50–8.03, p = 0.325). The indoor UV-LT collected a mean of 0.08 *An. coustani* per trap-night (RR = 0.89, 95% CI 0.18–4.47, p = 0.887) while no *An. coustani* were collected by indoor aspiration.

Outdoors, when data was aggregated to night of collection or day of collection (for aspiration), there were no differences in the means for the two outdoor locations (outdoor far and outdoor close) for either the CDC-LT, the UV-LT or HLCs for any of the species. The data sets for the two outdoor locations were therefore combined as outdoors in the daily mean analysis. Outdoor UV-LT collected significantly higher numbers of *Anopheles* mosquitoes across all species analysed (*An. funestus* mean = 1.69, RR = 5.18, 95% CI 2.68–10.00, p < 0.001; *An. arabiensis* mean = 0.22, RR = 15.64, 95% CI 1.97–124.36, p = 0.009; *An. coustani* mean = 3.74, RR = 11.65, 95% CI 5.18–26.20, p < 0.001) when compared to outdoor HLC. Outdoor CDC-LT also collected higher mosquitoes compared to outdoor HLC for all three species (*An. funestus* mean = 1.00, RR = 3.09, 95% CI 1.62–5.90, p < 0.001; *An. arabiensis* mean = 0.15, RR = 10.81, 95% CI 1.34–87.35, p = 0.026; *An. coustani* mean = 2.14, RR = 11.22, 95% CI 4.95–25.43, p < 0.001 (Table 2). For outdoor aspiration, significantly fewer *An. funestus* were collected per sampling effort compared to HLC (mean = 0.06, RR = 0.21, 95% CI 0.07–0.67, p = 0.008).

**Table 2:** Comparison of mean numbers of *Anopheles* mosquitoes caught by UV-LT, CDC-LT and aspiration to HLC

Species	Collection position	Collection method*	Mean daily trap catch	RR (95% CI)	P-value
*An. funestus*	Indoors	CDC-LT^a^	1.74 (1.02–2.45)	1.89 (1.07–3.34)	0.028
		UV-LT^b^	3.70 (2.59–4.82)	3.97 (2.28–6.92)	< 0.001
		Aspiration^c^	6.74 (4.69–8.78)	8.83 (4.72–16.52)	< 0.001
		HLC^d^ (Ref)	0.97 (0.61–1.39)	Ref	Ref
	Outdoors	CDC-LT^a^	1.00 (0.74–1.40)	3.09 (1.62–5.90)	< 0.001
		UV-LT^b^	1.69 (1.06–2.32)	5.18 (2.68–10.00)	< 0.001
		Aspiration^c^	0.06 (0.01–0.12)	0.21 (0.07–0.67)	0.008
		HLC^d^ (Ref)	0.37 (0.15–0.60)	Ref	Ref
*An. arabiensis*	Indoors	CDC_LT^a^	0.18 (0.06–0.29)	5.75 (1.20–27.48)	0.028
		UV-LT^a^	0.18 (0.07–0.30)	5.87 (1.22–28.34)	0.028
		Aspiration^ab^	0.10 (0.03–0.17)	3.38 (0.64–17.90)	0.152
		HLC^b^ (Ref)	0.03 (0–0.08)	Ref	Ref
	Outdoors	CDC-LT^ab^	0.15 (0.05–0.26)	10.81 (1.34–87.35)	0.026
		UV-LT^b^	0.22 (0.11–0.33)	15.64 (1.97–124.36)	0.009
		Aspiration^ac^	0.05 (0–0.11)	3.59 (0.38–34.28)	0.267
		HLC^c^ (Ref)	0.01 (0–0.04)	Ref	Ref
*An. coustani*	Indoors	CDC-LT^a^	0.29 (0–0.64)	2.01 (0.50–8.03)	0.325
		UV-LT^a^	0.08 (0.02–0.15)	0.89 (0.18–4.47)	0.887
		Aspiration	0	–	–
		HLC^a^ (Ref)	0.08 (0–0.18)	Ref	Ref
	Outdoors	CDC-LT^a^	2.14 (0.46–3.82)	11.22 (4.95–25.43)	< 0.001
		UV-LT^a^	3.74 (1.28–6.20)	11.65 (5.18–26.20)	< 0.001
		Aspiration^b^	0.23 (0.05–0.41)	1.25 (0.30–5.17)	0.755
		HLC^b^ (Ref)	0.29 (0–0.58)	Ref	Ref

*Post hoc comparison of the trapping methods. Methods bearing the same letter do not differ significantly at 5% level. Note that for *An. coustani* indoors, pairwise comparisons with aspiration could not be done as there were no females of this species collected by aspiration

All variables with the same letter implies that the trapping methods do not differ significantly at 5% level based on mean daily trap catch for each mosquito species. If two variables have different letters, they are significantly different at 5% level

Pairwise comparisons of mean densities of different *Anopheles* species by collection methods and location are presented in Additional file 1: Table S1. When a post hoc analysis was done to compare the performance in mean mosquito collection between traps, aspiration collected statistically more *An. funestus* indoors mean = 6.74, RR = 8.83, 95% CI 4.72–5.16.52, p < 0.001 than UV-LT which in turn collected significantly more *An. funestus* (mean = 1.74; RR = 1.89, 95% CI 1.07–3.34, *P* = 0.03) compared to indoor CDC-LT (mean = 3.70; RR = 3.97, 95% CI 2.28–6.92,* P* < 0.001).

Outdoors, UV-LT collected significantly more *An. funestus* (mean = 1.69, RR = 5.18, 95% CI 2.68–10.00, p < 0.001) compared to CDC-LT (mean = 1.00, RR = 3.09, 95% CI 1.62–5.90, p < 0.001), which collected significantly more *An. funestus* compared to outdoor aspiration (mean = 0.06, RR = 0.21, 95% CI 0.07–0.67, p = 0.008). Indoors, UV-LT and CDC-LT collected significantly higher numbers of *An. arabiensis* compared to HLC but no other pairwise comparisons were significantly different. Outdoors, UV-LT and CDC-LT collected significantly more *An. arabiensis* compared to HLC while UV-LT collected significantly more *An. arabiensis* compared to aspiration. Outdoor UV-LT and CDC-LT collected significantly more *An. coustani* compared to HLC and aspiration collections but there were no differences in mean numbers of *An. coustani* by the different trapping methods indoors (Table 2).

### Comparison of hourly mosquito collections by trapping method

**Fig. 1:**
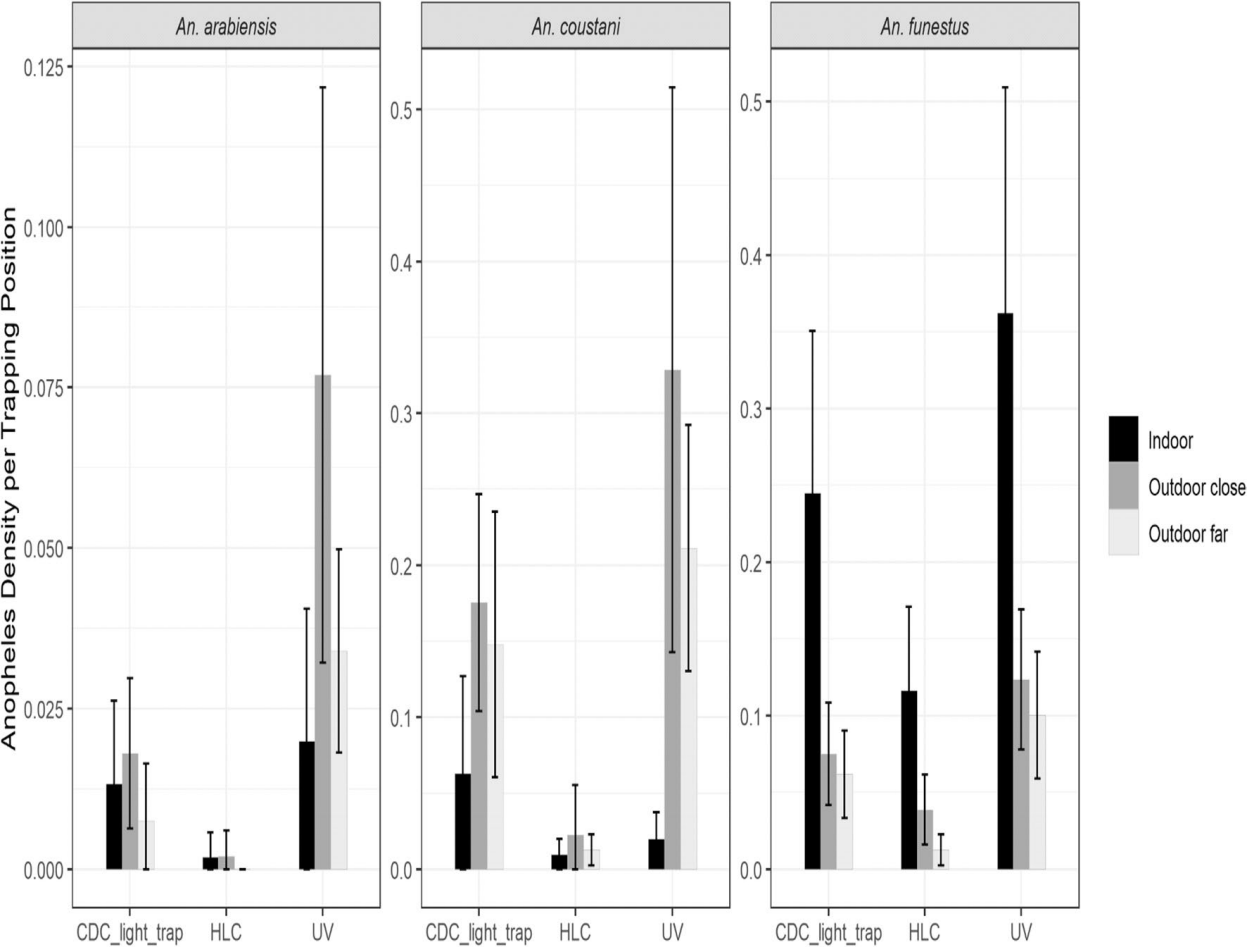
Comparison of UV-LT, CDC-LT and HLC at three different locations

**Fig. 2:**
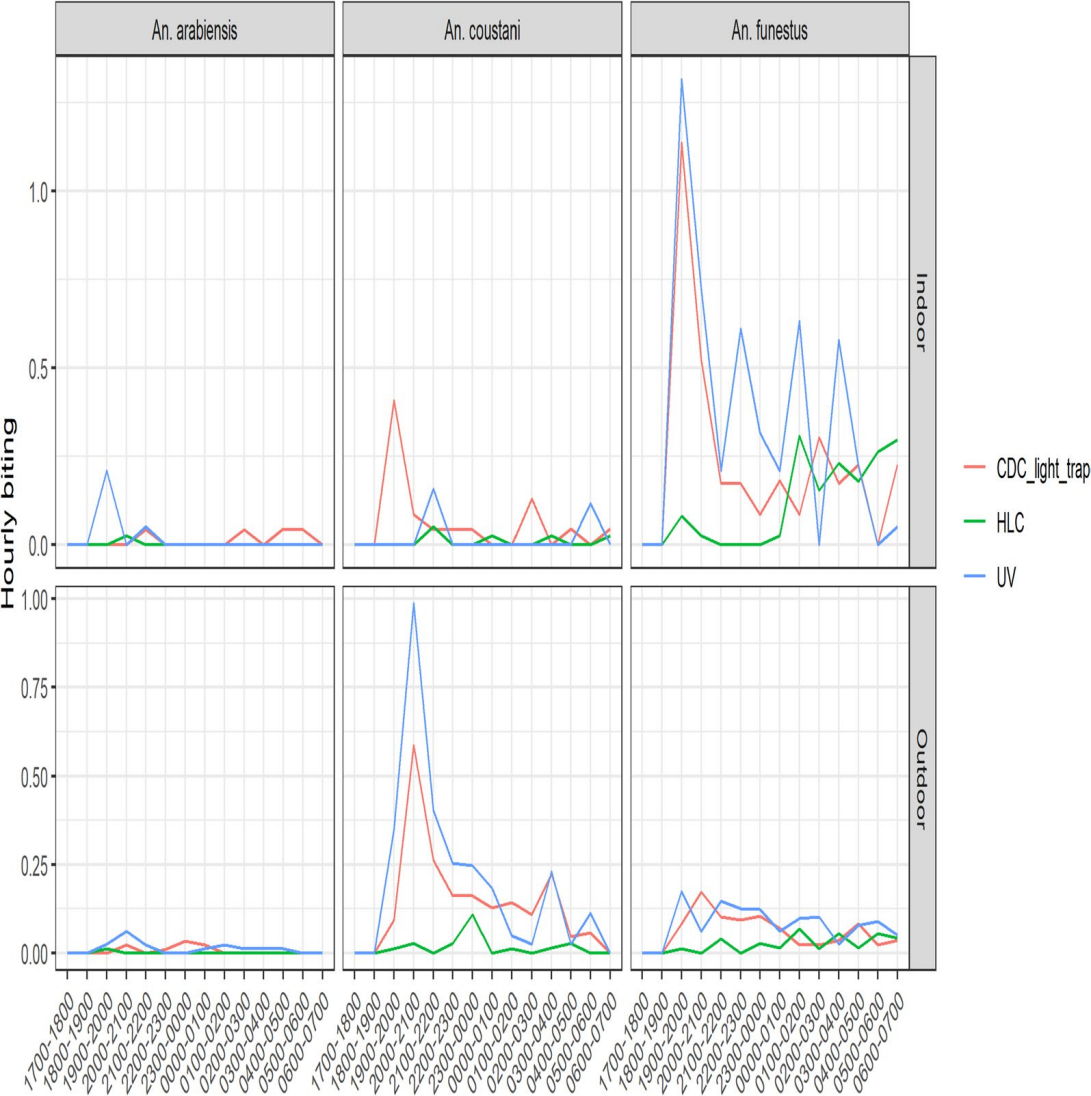
Comparison of hourly trap catches from indoor and outdoor locations

The mean number of mosquitoes captured by hour and by location using the three different collection methods are presented in Fig. 1. The hourly biting patterns are shown in Fig. 2. By HLC, biting by *An. funestus* was low from the start of collections until midnight when there was increased biting reaching a plateau that remained consistent throughout the remainder of the night. In contrast, a peak of activity for *An. funestus* was observed by both CDC-LT and UV-LT between 7 and 8 p.m. which diminished rapidly but activity was still observed throughout the night by both methods. Outdoors, *An. funestus* showed similar patterns although they were less distinct given the lower number of mosquitoes collected. For *An. coustani* outdoors, a peak in activity was observed by CDC-LT and UV-LT between 8 and 9 p.m. which declined rapidly though some activity was still observed throughout the night. Numbers of *An. coustani* collected by HLC outdoors or by any collection method indoors were too low to discern a pattern. Similarly, the numbers of *An. arabiensis* collected by the three methods both indoors and outdoors were too low to detect a clear pattern of activity throughout the night.

## Discussion

This study compared the efficiency of different trapping methods, placed at different locations around the peridomestic space to identify the most suitable method or set of methods to use as potential alternatives to HLCs*. Anopheles funestus* was predominantly caught resting indoors with aspiration being the most effective method of collection. Based on the mean numbers collected, UV-LT outperformed the CDC-LT in trapping *An. funestus* indoors and outdoors. The UV-LT also collected more *An. arabiensis* and *An. coustani* compared to the CDC-LT except for sampling *An. coustani* indoors; however, other than for *An. funestus* indoors, none of these observed differences were statistically significant. The UV-LT and CDC-LT trapped more mosquitoes than HLC both indoors and outdoors. Hourly biting rates in UV-LT and CDC-LT indicated different peaks in biting from that of HLC which raises questions about the physiological state and behaviour of mosquitoes captured by the different collection methods.

The observation of *An. funestus* as the primary vector collected during the evaluation of these trapping methods coupled with the fact that these mosquitoes were mostly captured indoors demonstrates the resilience in this vector species after years of high coverage of ITNs in the study area. *Anopheles funestus* reemerged [18] after almost being eliminated in the study area following the distribution of ITNs in 2000s [37]. Multiple research groups have reported resurgences of *An. funestus* despite sustained control efforts in multiple countries [18, 38]. The reemergence of *An. funestus* is likely associated with high levels of pyrethroid resistance that developed in this species [39, 40]. The fact that only indoor collected *An. funestus* were positive for sporozoites indicates that the bulk of malaria transmission in this area is likely propagated indoors by this species and complementary indoor vector control tools are needed to achieve malaria elimination.

All the *An*. *gambiae s.l*. caught by the different trapping methods were *An. arabiensis*. The predominance of *An. arabiensis* compared to *An. gambiae s.s*. following the scale up of ITNs was previously reported [19, 41–43] indicating that *An gambiae s.s*. has not responded in the same way as *An. funestus* despite the presence of phenotypic and genotypic resistance in *An. gambiae s.s* [44]. *Anopheles arabiensis* were mostly collected outdoors by light traps and aspiration from clay pots, consistent with the species’ exophilic and exophagic behaviour previously reported in in the region [45–47]. This likely has enabled them to avoid indoor deployed interventions, such as LLINs and IRS [14, 48, 49]. Despite not being detected in the current study, sporozoite positive *An. arabiensis* have been reported previously albeit at lower rates compared to *An. funestus* [18]. Given their tendency to feed and rest outdoors, *An. arabiensis* may contribute to residual transmission of malaria [50]. The presence of *An. coustani* in the peri-domestic space has been observed previously [51] but their importance for malaria transmission remains to be elucidated.

Comparison of different mosquito trapping methods indicates that mechanical aspirations indoors and UV-LT outdoors captured high numbers *An. funestus* mosquitoes. UV-LT performed well outdoors and indoors, second only to aspiration in the number of *An. funestus* mosquitoes collected indoors. UV-LTs generally collected more mosquitoes than CDC-LTs, although the difference was statistically significant only for the collection of *An. funestus* indoors and outdoors. It is possible that the efficacy of incandescent light in CDC-LTs may be affected by other light sources in the night including moonlight [52]. Also, mosquitoes have diverse response to different light spectra as previously reported where mosquito response to artificial light indicated that blue and green light is often more attractive than that in the yellow-orange and red regions of the visible spectrum [53, 54]. UV-LT is a largely unexplored trapping technique that could be useful for both indoor and outdoor trapping of mosquitoes especially when evaluating outdoor deployed vector control methods such as ATSBs as was recently done in Mali [55].

Fewer mosquitoes were collected by HLCs compared to both UV-LT and CDC-LT both indoors and outdoors. Previous comparisons of HLCs versus CDC LTs have resulted in diverse outcomes [56] with some indicating greater efficiency of HLCs [57, 58] and others indicating higher efficiency of CDC LTs [46, 59]. In western Kenya, HLCs were also less efficient in collecting the primary vectors compared to the Furvela tent trap, the host decoy trap, mosquito electrocuting traps and outdoor CDC light traps [52]. While HLCs are considered the gold standard for monitoring entomological measures of malaria transmission, the low numbers collected suggest they may underestimate entomological inoculation rates. The reason for the low numbers captured by HLCs is not clear as the collectors were provided adequate training and supervision. It is possible that light traps and aspiration collections capture more than just host-seeking mosquitoes [16, 56, 60].

Human landing catches remains the gold standard method for monitoring the abundance and host-seeking behaviour of mosquitoes because they elicit the natural host-seeking activity of malaria vectors using the same cues such as carbon dioxide, host odors, body heat and images. Other traps such as CDC light trap deploy light cues or artificial odors to attract mosquitoes and as such may not be used to accurately study the host-seeking activity of malaria vectors with the precision seen in HLC [61–64]. Furthermore, HLCs are easy to standardize and can be conducted in rural settings with limited access to electricity. However, HLCs are labour-intensive and potentially expose collectors to infectious mosquito bites. Therefore, CDC-LT, and less frequently UV-LT, are routinely used in monitoring *Anopheles* abundance during entomological surveillance. These traps are usually set up in the evening and left to run uninterrupted the whole night and therefore are unable to account for the specific hours at which mosquitoes were trapped as an indicator of host-seeking behaviour [12, 56, 65, 66]. Rotator light traps have been used to assess diel mosquito activity in studies of *Aedes* mosquitoes [67–69] and less frequently to monitor *Anopheles* activity [41, 61, 70]. In this study, CDC-LT and UV-LT bags were collected every hour through the night. Despite being labour-intensive and intrusive, this method allowed a direct comparison of the mosquito host-seeking behaviour patterns to those usually depicted by HLC. In western Kenya, previous HLC collections demonstrated a single peak in biting by *An. funestus* that extended from midnight until around 6 a.m. [71] similar to what was observed in this study. CDC-LT and UV-LT identified high mosquito activity early in the evening when people are unlikely to be under the protection of their bed nets. This differed from the HLC collections which is consistent with previous observations where biting was observed primarily when people were in bed and under their bed nets. Similar observations have been reported in the highlands of western Kenya [9] where it was suggested that transmission could occur at times when people were not under the protection of nets. However, the differences in collection times by the different methods raises questions about mosquito behaviour in the peridomestic space including those unrelated to host-seeking. Observations from this study suggest that while CDC-LTs and UVLTs may be useful as proxy indicators of the total mosquito population, they may not represent the host-seeking population of mosquitoes and care should be taken in interpreting the results of CDC-LTs and UVLTs as proxies for HLC in estimating EIRs. *An. arabiensis* densities were too low to derive any meaningful inferences on their behaviour indoors but like *An. coustani*, were observed to peak early in the evening outdoors.

Some limitations in this study included low number of *An. funestus* samples collected using HLC and limited number of *An. arabiensis* in all trapping methods therefore reducing the statistical power. Comparisons of the times of collection by the various methods were limited by the fact that some households refused entry while they were asleep and this may have biased the activity patterns of mosquitoes collected by CDC-LTs and UV-LTs. However, this was accounted for by limiting analyses of biting times to only those households with at least 12 hourly collections over the course of the night.

## Conclusion

*Anopheles funestus* was the predominant malaria vector in this study with lower numbers of *An. arabiensis* and *An. coustani*. This study indicates that aspiration, CDC-LTs and UV-LTs are efficient methods for trapping *Anopheles* mosquitoes indoors and outdoors and often collect more mosquitoes than HLCs. Although not statistically significant, UV-LTs generally collected more mosquitoes than CDC-LTs. Different trends in collection times were observed for *An. funestus* when collected by CDC-LT and UV-LTs compared to HLCs. This suggests that the different collection methods are capturing mosquitoes engaged in different behaviours throughout the night.

### Supplementary Information

**Additional file 1: Table S1.** Pairwise comparisons of means of different *Anopheles* species between collection methods.

## Data Availability

This is not applicable, however, the source document has been referenced.

## Abbreviations

CDC: Centers for disease control
ELISA: Enzyme linked immunosorbent assay
GVCR: Global Vector Control Response
HLC: Human landing catch
ID: Identification
IRS: Indoor residual spray
LLINs: Long lasting insecticidal nets
KEMRI: Kenya Medical Research Institute
PCR: Polymerase chain reaction
CDC-LTs: Center for disease control light traps
UV-LTs: Ultra violet light traps
WHO: World Health Organization
