# Supplementary material for: Comparison of different trapping methods to collect malaria vectors indoors and outdoors in western Kenya
Source: Malar J. 2024 Mar 16;23:81. doi: 10.1186/s12936-024-04907-0 (PMC10943837; doi:10.1186/s12936-024-04907-0)
Supplement: Supplementary file 1 — Additional file 1: Table S1. Pairwise comparisons of means of different Anopheles species between collection methods. [file 12936_2024_4907_MOESM1_ESM.docx]

| **Table S1: Pairwise comparisons of means of different *Anopheles* species between collection methods** | | | | | | |
| --- | --- | --- | --- | --- | --- | --- |
| **Method** | **Value** | **Reference** | **Reference Value** | **RR (95% CI)** | **Z-Score** | **P-Value** |
| *An. funestus*, Indoors | | | | | | |
| CDC-LT | 1.74 | HLC | 0.97 | 1.89 (1.07 - 3.34) | 2.201 | 0.028 |
| CDC-LT | 1.74 | Aspiration | 6.74 | 0.21 (0.12 - 0.38) | -5.314 | <0.001 |
| CDC-LT | 1.74 | UV-LT | 3.70 | 0.48 (0.29 - 0.78) | -2.981 | 0.003 |
| UV-LT | 3.70 | HLC | 0.97 | 3.97 (2.28 - 6.92) | 4.861 | <0.001 |
| UV-LT | 3.70 | Aspiration | 6.74 | 0.45 (0.26 - 0.78) | -2.836 | 0.005 |
| Aspiration | 6.74 | HLC | 0.97 | 8.83 (4.72 - 16.52) | 6.811 | <0.001 |
| *An. funestus*, Outdoors | | | | | | |
| CDC-LT | 1.00 | HLC | 0.37 | 3.09 (1.62 - 5.90) | 3.420 | 0.001 |
| CDC-LT | 1.00 | Aspiration | 0.06 | 14.80 (4.86 - 45.07) | 4.744 | <0.001 |
| CDC-LT | 1.00 | UV-LT | 1.69 | 0.60 (0.36 – 1.00) | -1.969 | 0.049 |
| UV-LT | 1.69 | HLC | 0.37 | 5.18 (2.68 - 10.00) | 4.893 | <0.001 |
| UV-LT | 1.69 | Aspiration | 0.06 | 24.79 (8.15 - 75.41) | 5.657 | <0.001 |
| Aspiration | 0.06 | HLC | 0.37 | 0.21 (0.07–0.67) | -2.635 | 0.008 |
| *An. arabiensis*, Indoors | | | | | | |
| CDC-LT | 0.18 | HLC | 0.03 | 5.75 (1.20 - 27.48) | 2.191 | 0.028 |
| CDC-LT | 0.18 | Aspiration | 0.10 | 1.70 (0.60 - 4.83) | 0.996 | 0.319 |
| CDC-LT | 0.18 | UV-LT | 0.18 | 0.98 (0.40 - 2.40) | -0.047 | 0.963 |
| UV-LT | 0.18 | HLC | 0.03 | 5.87 (1.22 - 28.34) | 2.205 | 0.028 |
| UV-LT | 0.18 | Aspiration | 0.10 | 1.74 (0.60 - 5.01) | 1.020 | 0.308 |
| Aspiration | 0.10 | HLC | 0.03 | 3.38 (0.64 - 17.90) | 1.434 | 0.152 |
| *An. arabiensis*, Outdoors | | | | | | |
| CDC-LT | 0.15 | HLC | 0.01 | 10.81 (1.34 - 87.35) | 2.232 | 0.026 |
| CDC-LT | 0.15 | Aspiration | 0.05 | 3.01 (0.87 - 10.36) | 1.747 | 0.081 |
| CDC-LT | 0.15 | UV-LT | 0.22 | 0.69 (0.30 - 1.62) | -0.852 | 0.394 |
| UV-LT | 0.22 | HLC | 0.01 | 15.64 (1.97 - 124.36) | 2.599 | 0.009 |
| UV-LT | 0.22 | Aspiration | 0.05 | 4.35 (1.31 - 14.49) | 2.398 | 0.017 |
| Aspiration | 0.05 | HLC | 0.01 | 3.59 (0.38 - 34.28) | 1.111 | 0.267 |
| *An. coustani*, Indoors | | | | | | |
| CDC-LT | 0.29 | HLC | 0.08 | 2.01 (0.50 - 8.03) | 0.984 | 0.325 |
| CDC-LT | 0.29 | Aspiration | 0 | - | - | - |
| CDC-LT | 0.29 | UV-LT | 0.08 | 2.26 (0.63 - 8.06) | 1.251 | 0.211 |
| UV-LT | 0.08 | HLC | 0.08 | 0.89 (0.18 - 4.47) | -0.142 | 0.887 |
| UV-LT | 0.08 | Aspiration | 0 | - | - | - |
| Aspiration | 0 | HLC | 0.08 | - | - | - |
| *An. coustani*, Outdoors | | | | | | |
| CDC-LT | 2.14 | HLC | 0.29 | 11.22 (4.95 - 25.43) | 5.792 | <0.001 |
| CDC-LT | 2.14 | Aspiration | 0.23 | 8.96 (2.39 - 33.54) | 3.254 | 0.001 |
| CDC-LT | 2.14 | UV-LT | 3.74 | 0.96 (0.54 - 1.73) | -0.126 | 0.900 |
| UV-LT | 3.74 | HLC | 0.29 | 11.65 (5.18 - 26.20) | 5.938 | <0.001 |
| UV-LT | 3.74 | Aspiration | 0.23 | 9.30 (2.54 - 34.11) | 3.363 | 0.001 |
| Aspiration | 0.23 | HLC | 0.29 | 1.25 (0.30 - 5.17) | 0.311 | 0.755 |
